# Composition and Ecological Roles of the Core Microbiome along the Abyssal-Hadal Transition Zone Sediments of the Mariana Trench

**DOI:** 10.1128/spectrum.01988-21

**Published:** 2022-06-07

**Authors:** Hongmei Jing, Xiang Xiao, Yue Zhang, Zhiyong Li, Huahua Jian, Yingfeng Luo, Zhuang Han

**Affiliations:** a Chinese Academy of Sciences (CAS) Key Laboratory for Experimental Study under Deep-Sea Extreme Conditions, Institute of Deep-Sea Science and Engineering, Chinese Academy of Sciences, Sanya, China; b State Key Laboratory of Ocean Engineering, School of Naval Architecture, Ocean and Civil Engineering, Shanghai Jiao Tong University, Shanghai, China; c State Key Laboratory of Microbial Metabolism, Joint International Research Laboratory of Metabolic and Developmental Sciences, School of Life Sciences and Biotechnology, Shanghai Jiao Tong University, Shanghai, China; d CAS Key Laboratory of Genome Sciences and Information, Beijing Institute of Genomics, Chinese Academy of Sciences, Beijing, China; e Southern Marine Science and Engineering Guangdong Laboratory, ZhuHai, China; f Hong Kong University of Science and Technology (HKUST)-CAS Sanya Joint Laboratory of Marine Science Research, Chinese Academy of Sciences, Sanya, China; University of Mississippi

**Keywords:** Mariana Trench, metagenomics, core microbiome, abyssal-hadal transition zone

## Abstract

The unique geological features of hadal trenches are known to influence both the structure and ecological function of microbial communities. It is also well known that heterotrophs and chemoautotrophs dominate the hadal and abyssal pelagic zones, respectively. Here, a metagenomic investigation was conducted on sediment samples obtained from the abyssal-hadal transition zone in the Mariana Trench to gain a better understanding of the general diversity and potential function of the core microbiome in this zone. A high level of cosmopolitanism existed in the core microbiome referred from a high community similarity among different stations. Niche differentiation along the fine-scale of different sediment layers was observed, especially for major archaeal groups, largely due to sediment depth and the source of organic matter. A prevalence of nitrogen biogeochemical cycles driven by various nitrifying groups with the capability of dark carbon fixation in the abyssal-hadal biosphere was also demonstrated. The predominance of heterotrophic over chemolithoautotrophic pathways in this transition zone was found, and a high abundance of genes related to respiration and carbon fixation (i.e., the intact Calvin and rTCA cycles) were detected as well, which might reflect the intensive microbial activities known to occur in this deep biosphere. The presence of those metabolic processes and associated microbes were reflected by functional and genetic markers generated from the metagenomic data in the current study. However, their roles and contributions to the nitrogen/carbon biogeochemical cycles and flux in the abyssal-hadal transition zone still need further analysis.

**IMPORTANCE** The Mariana Trench is the deepest oceanic region on earth, its microbial ecological exploration has become feasible with the rapid progress of submersible and metagenomic sequencing. We investigated the community compositions and metabolic functions of the core microbiome along the abyssal-hadal transition zone of the Mariana Trench, although most studies by far were focused on the pelagic zone. We found a predominance of heterotrophic groups and related metabolic pathways, which were closely associated with nitrogen biogeochemical cycles driven by various nitrifying groups with the capability of dark carbon fixation.

## INTRODUCTION

The deep-sea environment is unique on earth as it is characterized by being in near-total darkness with high hydrostatic pressure, a low average temperature (of ~4°C), and a low supply of organic matter (i.e., 1 to 10 mmol C m^−2^yr^−1^) (1). Among these different environmental factors, the supply of organic matter (from both vertical fluxes and lateral transport from the continental shelves) is essential to support deep-sea ecosystems (2). It has been estimated that deep-sea sediments act as a huge reservoir of organic carbon with ~13% of the total global bacteria living in the upper 10 cm (1, 3), and the cell abundance decreased from 10^6^ cells/cm^3^ at seafloor surface to 10^3^ cells/cm^3^ at 10 m along with the different sediment layers (4). This suggests that a spatial variation of the microbial communities with sediment depths exists even in microniches in the different sediment layers, and so it would be advantageous to verify the niche separation of microbes in terms of fine-scale heterogeneity.

Trenches are the deepest oceanic areas, and these feature an extremely high hydrostatic pressure (e.g., >60 MPa) and isolated hydrotopographical conditions (5). Owing to the funnel structure of trenches, the sediments accumulate particularly along the trench axis and vary in terms of quality and quantity with depths (6). Moreover, *in situ* chemoautotrophic and mixotrophic microbial processes provide additional sources of organic carbon, and together these adjust any imbalance that occurs between the input of organic carbon through vertical fluxes and its biological consumption (7). The Hadal zone is a relatively nutrient-enriched environment, where a higher abundance of heterotrophic microorganisms (6) has been detected than in the adjacent abyssal plain (8). For these reasons, characterizing the composition, distribution, and metabolic pathways of the microbial communities in the different sediment niches will provide important insights into the ecological and biogeochemical functioning of this extreme ecosystem.

The Mariana Trench is the deepest oceanic region on Earth, and the deepest part of this trench is called the Challenger Deep. It is geographically and hydrotopographically isolated from other trenches in the Western Pacific (6). The steep slope, narrow geomorphology, and slow trench current provide a steady supply and the occasional input of sinking and suspended organic matter (6, 9, 10), as well as a high microbial carbon turnover rate (11). So far, most of the microbial ecological studies conducted in the Mariana Trench have been focused on water samples (6, 12–16). In these studies, the distribution and abundance of the entire microbial community (and specifically the nitrifiers) are distinct between the hadal and abyssal waters, perhaps due to the diverse fluxes of electron donors at the different depths (6). Therefore, the abyssal-hadal boundary that is known to exist for benthic amphipoda (17) is also applicable to the vertical distribution of microbes. On the other hand, so far only limited information is available regarding the composition and ecological function of the microbes that inhabit the sediments of the abyssal-hadal transition zone in the Mariana Trench (11, 18). Whether a core microbial community exists at all among the various sampling stations in the abyssal-hadal transition zone, as well as its potential metabolic functions, are still largely unknown. A core microbiome represents those genomes or genetic markers common to all the samples studied and is critical to the genetic functions and composition of the microbial communities (19). Therefore, it is crucial to identify the community structures and ecological functions of the core microbiome in this deep oceanic biosphere.

In this study, sediment samples were collected from eight sampling stations along the abyssal-hadal transition zone (i.e., ~5,455 to 6,707 m) in the Mariana Trench. The top three layers (0 to 6 cm, 6 to 12 cm, and 12 to 18 cm) of each sediment sample were investigated with metagenomics to elucidate the community structure and potential functions of microbes along the abyssal-hadal transition zone with geographical scales ranging from just a few centimeters to hundreds of kilometers. Our core microbiome studies also allowed us to identify and define the relative stable (i.e., core) communities that contribute to important biological functions in the abyssal-hadal transition zone.

## RESULTS

### Geochemical characterization of the sediments.

**FIG 1:**
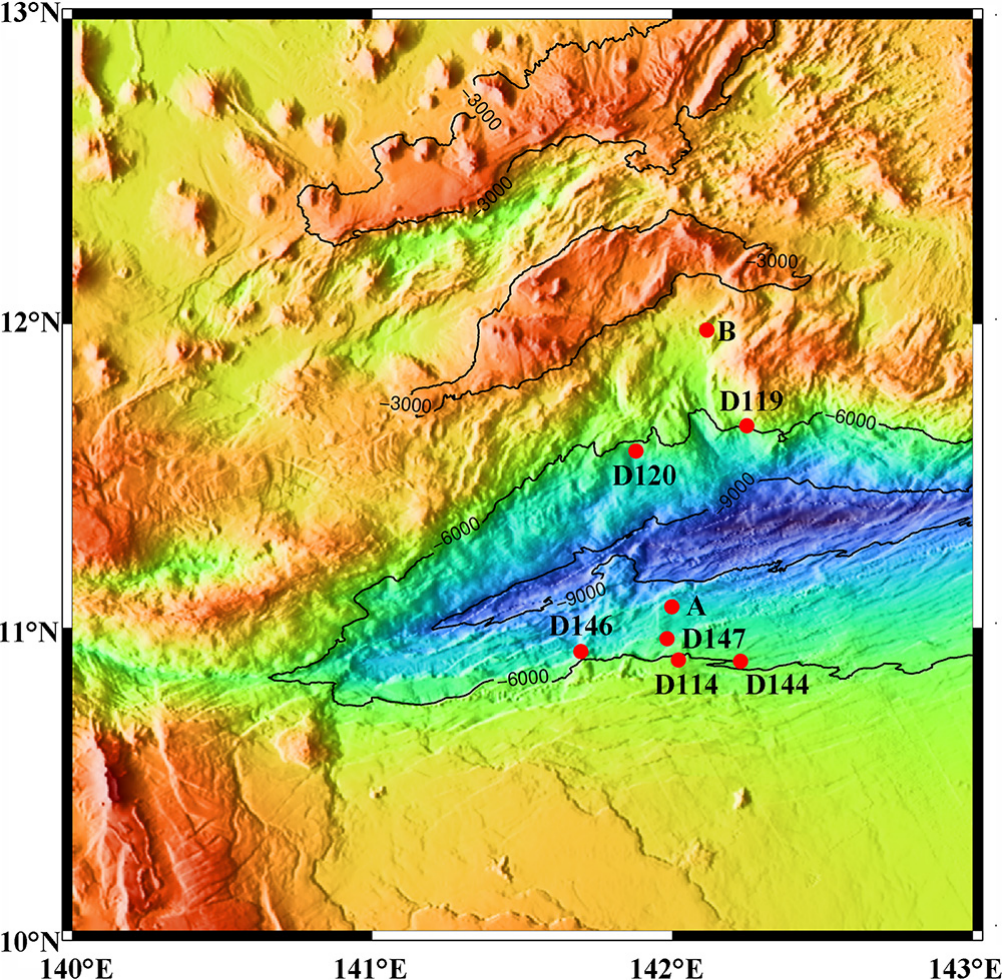
Location of the sampling stations in the Mariana Trench.

We investigated sediment core samples collected from eight stations across the abyssal-hadal zone (5,455 to 6,707 m) along the slopes of the Challenger Deep of the Mariana Trench (Fig. 1). The nutrient content of each sample was analyzed and calculated based on the dry weight of the sediment (Table 1). The concentration of NH_4_^+^-N was significantly higher than that of NO_3_-N in all the samples except Station D147 where the lowest concentrations of NH_4_^+^-N and NO_3_-N were detected. All the samples exhibited a clear pattern of TC > TP > TN, and the highest C/N occurred at Station B. At Stations B and D119, the concentration of all the nutrients decreased with the increase in sediment depth, except for the concentration of NO_3_-N at Station B and δ^15^N at Station D119, which had lower values shown in the deeper layers. With regard to Stations D144 and D147, all the nutrients (apart from C/N and TP), were at the lowest concentration in the surface layer (0 to 6 cm) and then increased with sediment depth. At Station D144, the highest concentration of TP occurred in the deepest layer (12 to 18 cm), whereas at Station B, the highest concentrations of TC, TN, C/N, and moisture were found in the surface layer. The values of δ^13^C and δ^15^N ranged from −26.41‰ to −22.81‰ and from 4.05‰ to 7.67‰, respectively. The highest values of δ^13^C and δ^15^N were detected in the middle layer (6 to 12 cm) at Station D147, whereas the lowest values of both were found at the middle layer of Station D119 and the surface layer of Station D146, respectively.

**TABLE 1:** Geochemical characteristics of the sediment samples collected from the Challenger Deep in the Mariana Trench

Stations	Longitude (E°)	Latitude (N°)	Depth (m)	Layers (cm)	TP (mg/kg)	TN (mg/kg)	TC (mg/kg)	C/N	δ^13^C (‰)	δ^15^N (‰)	NO_3_-N (mg/kg)	NH_4_-N (mg/kg)	Moisture content (%)
A	141.99	11.07	5455	0–6	NA	NA	NA	NA	NA	NA	NA	NA	NA
				0–6	1114.78	458	4500	9.79	−23.06	5.79	10.63	74.71	3.28
B	142.12	11.98	5481	6–12	1134.65	271	1500	5.69	−26.12	6.28	10.64	43.95	1.48
				12–18	468.93	348	2900	8.19	−23.25	6.59	4.33	58.62	1.74
D114	142.02	10.89	5482	0–6	NA	NA	NA	NA	NA	NA	NA	NA	NA
				6–12	NA	NA	NA	NA	NA	NA	NA	NA	NA
D119	142.25	11.67	6014	0–6	718.02	349	2500	7.42	−23.45	7.01	7.45	87.90	2.67
				6–12	521.31	220	1200	5.37	−26.41	5.10	7.89	50.83	0.84
				0–6	1026.97	240	1930	7.97	−25.18	5.42	6.02	47.51	1.59
D144	142.23	10.89	6333	6–12	1230.83	260	2070	8.18	−24.41	7.59	3.41	49.08	1.56
				12–18	1334.43	380	2930	7.66	−23.48	7.41	6.74	62.71	1.71
D146	141.70	10.92	6676	0–6	1113.41	322	2500	7.65	−23.69	4.05	9.91	70.07	2.99
D147	141.98	10.96	6693	0–6	1237.28	360	2670	7.37	−23.65	6.23	5.91	5.42	2.34
				6–12	1075.28	420	3030	7.16	−22.81	7.67	6.69	7.79	2.65
D120	141.88	11.58	6707	0–6	NA	NA	NA	NA	NA	NA	NA	NA	NA

### Core communities.

After trimming and assembly, a total of 14,918,045 contigs (>500 bp) were obtained, ranging from 650,818 to 1,218,486 contigs in each sample (Table 2). The average size of each contig was >817.60 bp and the largest contig was 143,936 bp. The minimum values for the predicted open reading frames (ORFs) and the total number of functional genes annotated in KEGG were 958,661 and 1,295,523, respectively.

**TABLE 2:** Basic information obtained from the metagenomic sequencing analysis

Samples^*a*^	Clean base (G)	No. of contig (>500bp)	Average size of contig (bp)	Largest contig (bp)	GC content (%)	Predicted ORFs	Total functional genes annotated in KEGG	Prokaryote /eukaryote	Bacteria /archaea
A_0_6	18.65	875,980	838	58,358	59.39	1,237,123	1,742,501	3.11	8.09
B_0_6	22.39	1,153,295	831	72,936	58.14	1,603,879	1,814,358	3.09	7.33
B_6_12	20.44	857,832	837	143,936	57.59	1,195,553	1,629,998	2.81	6.69
B_12_18	22.87	960,460	876	65,856	59.20	1,378,800	1,709,958	3.14	6.69
D114_0_6	22.38	1,189,823	817	56,294	59.80	1,632,839	1,821,091	2.78	9.00
D114_6_12	20.65	969,483	859	57,907	59.76	1,372,243	1,824,514	2.97	10.11
D119_0_6	21.79	1,026,915	836	43,575	58.09	1,434,069	1,855,670	2.88	9.00
D119_6_12	18.92	910,346	838	49,192	58.73	1,279,761	1,804,002	3.04	8.90
D144_0_6	21.02	1,021,121	860	51,400	59.80	1,456,968	1,789,263	2.91	9.00
D144_6_12	20.76	891,676	899	68,895	59.31	1,305,375	1,542,997	2.80	6.14
D144_12_18	14.70	650,818	913	113,024	59.76	958,661	1,295,523	2.79	7.33
D146_0_6	22.33	1,117,034	852	71,854	57.55	1,582,156	1,700,754	3.01	6.69
D147_0_6	24.03	1,052,633	853	61,437	58.68	1,491,949	1,750,158	2.98	5.25
D147_6_12	23.11	1,022,143	939	106,953	58.71	1,517,260	1,362,982	2.90	6.07
D120_0_6	27.88	1,218,486	905	78,955	58.89	1,779,131	1,763,186	2.89	7.33

a A, B, D114, etc. refer to the sampling site; 0_6, 6_12, 12_18 refer to the sampling depth.

**FIG 2:**
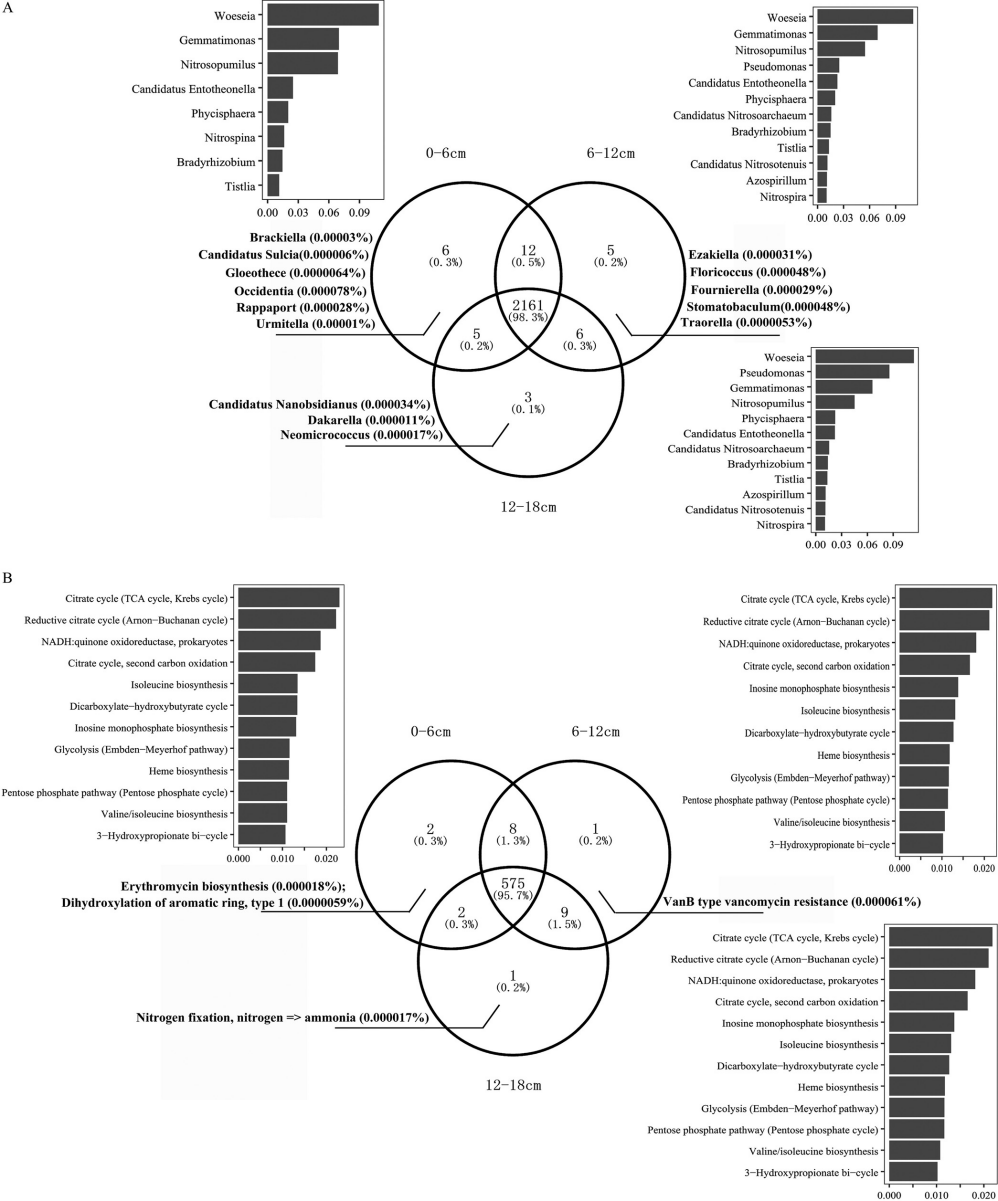
Venn diagram showing the overlap of prokaryotic sequences at the genus level (A) and the metabolic pathways at the module level based on the KEGG database (B) for the sediment samples collected at depths of 0 to 6 cm, 6 to 12 cm, and 12 to 18 cm in the Mariana Trench. Singletons were removed before analysis and both major and unique sequences were annotated.

The core microbiome was demonstrated in the Venn diagram, with 2,161 taxonomic sequences identified, which accounted for 98.3% of the integrated sequences from all samples (Fig. 2A). The microbial identities were determined by screening small subunit (SSU) rRNAs from the assembled contigs in each sample. *Woeseia*, *Gemmatimonas,* and *Nitrosopumilus* were the dominant prokaryotic genera within the core microbiome at all depths. In addition, Pseudomonas was also dominant in the 12 to 18 cm samples. Regarding unique sequences, there are twice as many on the surface (0.3%) as that in the deep (0.1%) sediments.

We also demonstrated that there were 575 core metabolic pathways in the identified core microbiome, and these collectively took up 95.7% of the integrated metabolic pathways from the sediment samples at all the depths (Fig. 2B). The dominant functional categories included the carbon cycling pathways, especially the TCA and rTCA cycles. The unique metabolic pathways among the different sediment depths ranged from ~0.2 to 0.3% and these were associated with erythromycin biosynthesis in the surface layer, vancomycin resistance in the middle layer, and nitrogen fixation in the deepest sediment layer.

### Community composition and impacting parameters.

**FIG 3:**
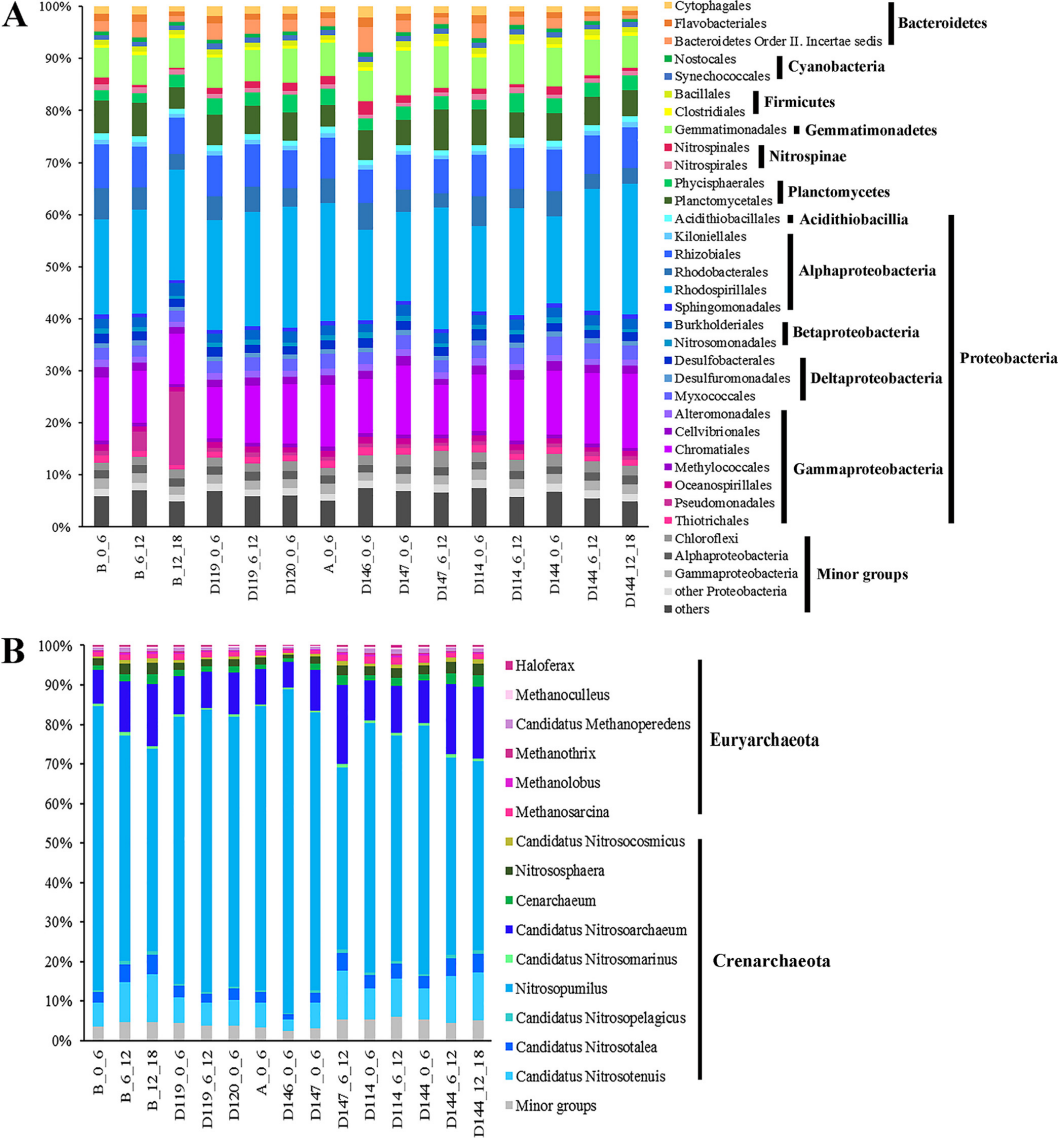
The community composition of the core microbiome is based on the top 30 orders of bacteria (A) and the top 15 genera of archaea (B). In both cases, group abundance <1% was treated as a minor group.

The community composition of the core microbiome was further analyzed. Bacteria accounted for a higher portion (i.e., 84.2 to 91.3%) than archaea (i.e., 8.7 to 15.8%) in all the samples excluding minor groups (i.e., <1%) (Fig. 3A). The highest relative abundance was detected at D114_6_12 for bacteria, and at D147_0_6 for archaea. Among the six bacterial phyla, Proteobacteria were in the majority (52.5 to 65.9%), and these exhibited a higher abundance in the deep layer of each sample. Rhodospirillales, Chromatiales, and Rhizobiales were the major orders of Proteobacteria found, and together their total proportions between the deepest and surface layers had no significant difference. With regard to the remaining bacterial phyla, Gemmationadales (6 to 7%) and Plantomycetales (4 to 8%) were distributed in all the samples without significant difference.

The main archaeal phyla detected were Crenarchaeota (89.2 to 95.6%) and Euryarchaeota (2.0 to 4.8%) (Fig. 3B). Within the dominant Crenarchaeota, there were *Nitrosopumilus* (46.1 to 82.0%), *Nitrosoarchaeum* (6.7 to 20.2%), and *Nitrosotenuis* (2.8 to 12.2%), whereas the Euryarchaeota was dominated by *Methanosarcina* (0.9 to 1.9%). The relative abundance of *Nitrosopumilus* decreased with an increase in sediment depth, whereas the opposite trend occurred for *Nitrosoarchaeum* and *Nitrosotenuis*, as more of these were found in the deep layer.

**FIG 4:**
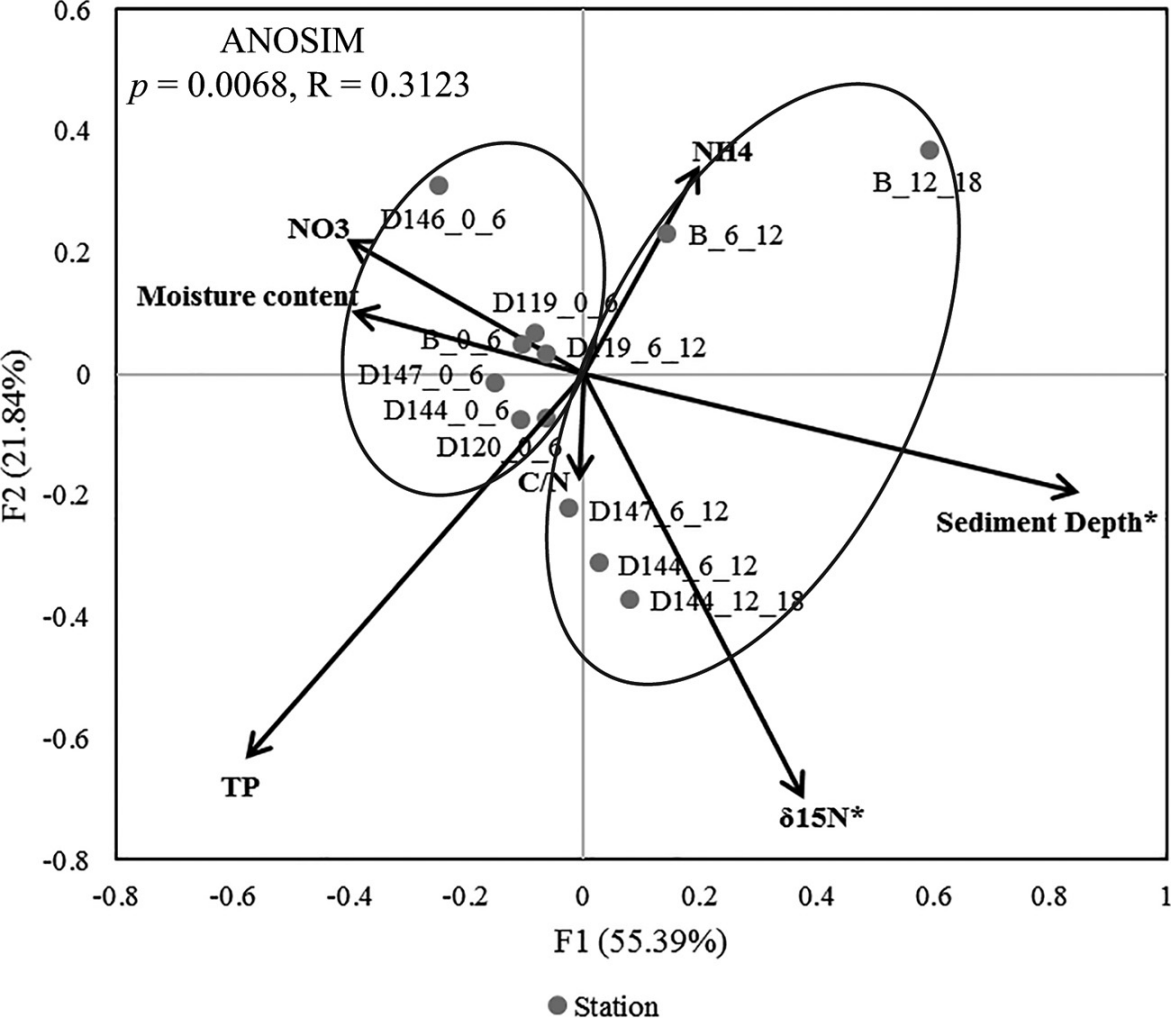
Redundancy analysis integrating the environmental parameters and the relative abundance of prokaryotic taxa at the order level in the different sediment samples (*, *P* < 0.05).

Redundancy analysis (RDA) based on the microbial communities at the class level demonstrated that sediment depth and δ^15^N were the key environmental parameters that significantly influenced the prokaryotic community structure (*P* < 0.05, Fig. 4). The first two axes together explained 77.2% of the total variance. In general, the former axis had a higher moisture content and NO_3_-N concentration, whereas the latter axis showed higher δ^15^N values. Samples from the surface and deeper layers formed two distinct clusters (analysis of similarities [ANOSIM], *P* < 0.01), consisting of the patterns shown in the nMDS plot based on the bacterial (Fig. S1A) and archaeal (Fig. S1B) assemblages at the order level. In terms of the average *α*-diversity (Shannon and Chao1) of the bacterial community at the order level, the surface cluster exhibited a significantly higher diversity (*P* < 0.05) than the deeper cluster (Fig. S1C). In addition, the average *α*-diversity of the bacterial communities (Fig. S1C and E) was much higher than that of the archaeal communities (Fig. S1D and F), and the latter also exhibited higher diversity in the surface cluster.

### Major metabolic pathways.

A total of 25,406,955 functional genes were identified in the core microbiome and were assigned to selected subsets of KEGG categories to identify potential functions in the predicted ORFs. Functional categories related to the biogeochemical cycling of elements were distributed differently among the samples. For example, the relative abundance of the denitrification genes (*nor*B and *nor*C) was significantly higher at Station D144 (*P* < 0.05), whereas genes related to nitrification (*P* < 0.05), nitrogen fixation, and denitrification were more abundant at Station D147. In addition, the key genes involved in sulfur oxidation (*P* < 0.05), dissimilatory sulfate reduction, 3-HP, and HP/HB had a higher abundance at Stations D119, A, and D146 than at other stations.

**FIG 5:**
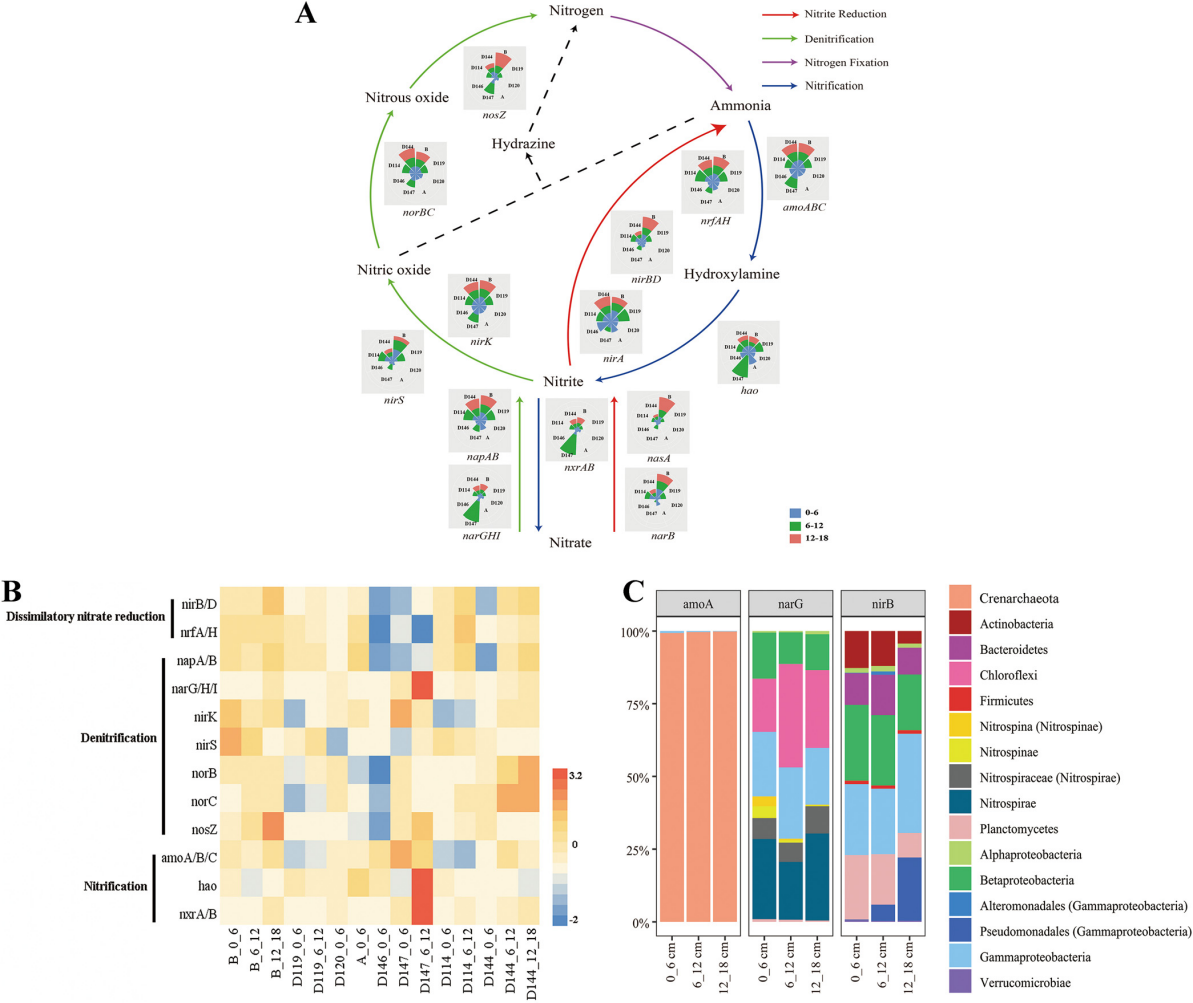
(A) Schematic with pie charts showing the relative abundance of genes involved in the main metabolic pathways of nitrogen in the core microbiome. The abundance was calculated by dividing the sum of the coverage of a particular gene in an individual sample, by the sum of the coverage of that gene in all the samples. (B) The absolute abundance of each gene was shown by z-score heatmap boxes indicated in the nitrogen cycle. (C) The microbial taxa of typical genes and their relative abundance in the different depths of each region.

Three nutrient cycling categories (i.e., nitrogen, carbohydrate, and sulfur metabolism) were analyzed in further detail. With regard to nitrogen metabolism, five pathways (i.e., dissimilatory and assimilatory nitrate reduction, denitrification, nitrification, and anammox) were all detected although the anammox pathway was incomplete due to a lack of the *hzs* and *hdh* genes (Fig. 5A). Of the genes involved in nitrogen fixation, only *nif*H was detected and this was at a very low abundance. Denitrification (*nar*, *nir*, *nor,* and *nos*), nitrification (*nxr*) and N-reduction (*nir* and *nrf*) genes were also significantly higher in the deep layers than at the surface (*P* < 0.05). Ammonia-oxidizing genes (*amo*A, *amo*B, and *amo*C) were most abundant in the surface sediment at Station D147_0_6 (Fig. 5B). *amo*A-affiliated microbial taxa were almost exclusively Crenarchaeota with slightly Gammaproteobacteria, *nar*G was associated with Chloroflexi, Nitrospina, Nitrospira, Planctomycetes, Betaproteobacteria, and Gammaproteobacteria (Pseudomonadales), and *nir*B affiliated with Planctomycetes, Actinobacteria, Bacteroidetes, Betaproteobacteria, and Gammaproteobacteria (Alteromonadales, Chromatiales, Pseudomonadales and Verrucomicrobiae) (Fig. 5C).

**FIG 6:**
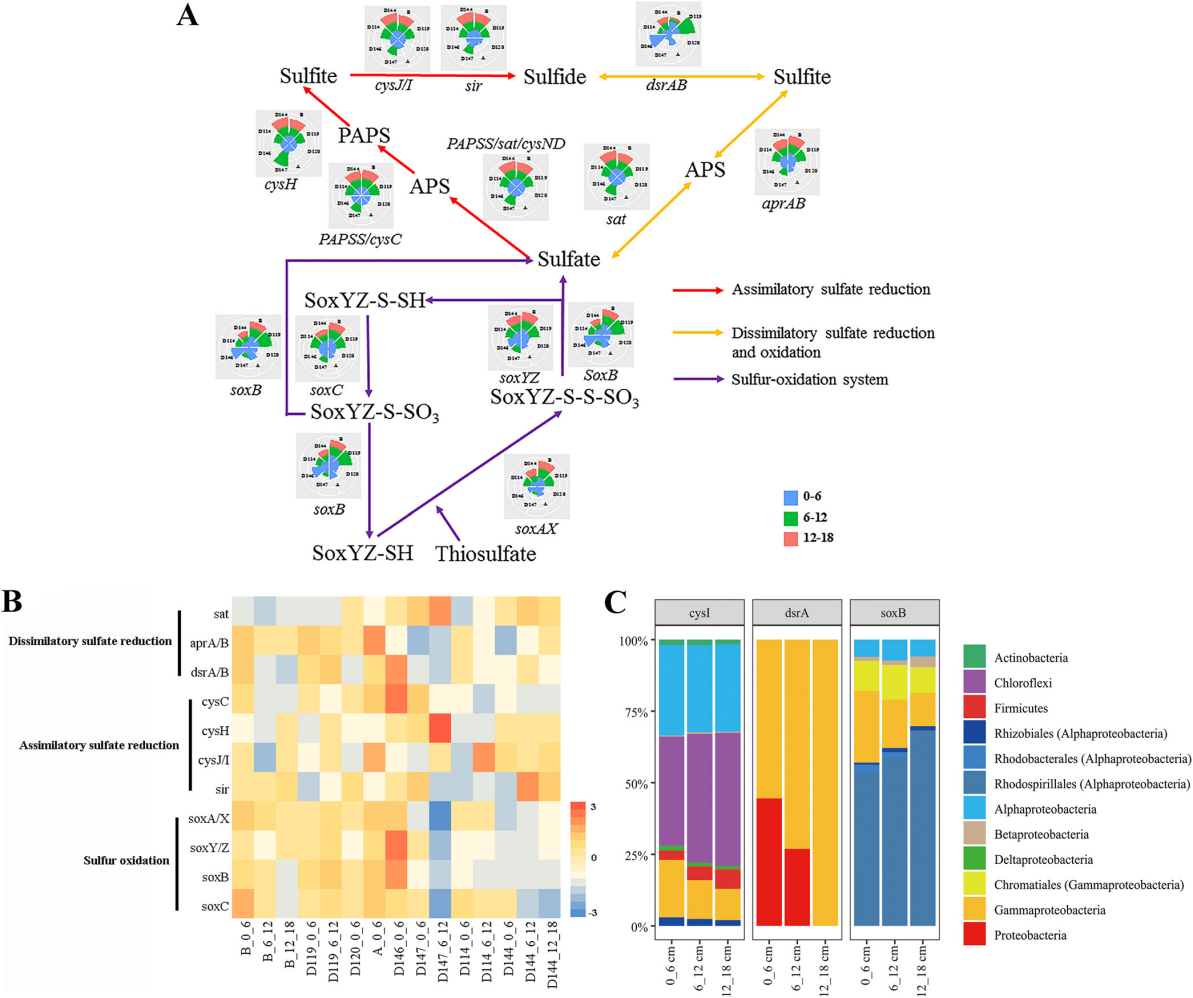
(A) Schematic with pie charts showing the relative abundance of genes involved in the main sulfur metabolic pathways in the core microbiome. The abundance was calculated by dividing the sum of the coverage of a particular gene in an individual sample, by the sum of the coverage of that gene in all the samples. (B) The absolute abundance of each gene was shown by z-score heatmap boxes indicated in the sulfur cycle. (C) The microbial taxa of typical genes and their relative abundance in the different depths of each region.

The genes involved in sulfur cycling (i.e., assimilatory sulfate reduction, dissimilatory sulfate reduction and oxidation, and the SOX system), were also detected (Fig. 6A). A higher abundance of genes involved in dissimilatory sulfate reduction (*dsr*A and *dsr*B) and the SOX system (*sox*) was found in the surface samples. The abundance of genes related to the SOX system decreased with depth, as they were higher in the surface layer than in the deep layers (*P* < 0.05) (Fig. 6B). *cys*I affiliated with microbial taxa of Chloroflexi and Alpha/Gamma/Deltaproteobacteria, *dsr*A associated with Proteobacteria, especially Gammaproteobacteria, *sox*B affiliated taxa were predominated by Alphaproteobacteria (Rhodospirillales, Rhizobiales, and Rhodospirillales) and low proportion of Gammaproteobacteria (Chromatiales).

**FIG 7:**
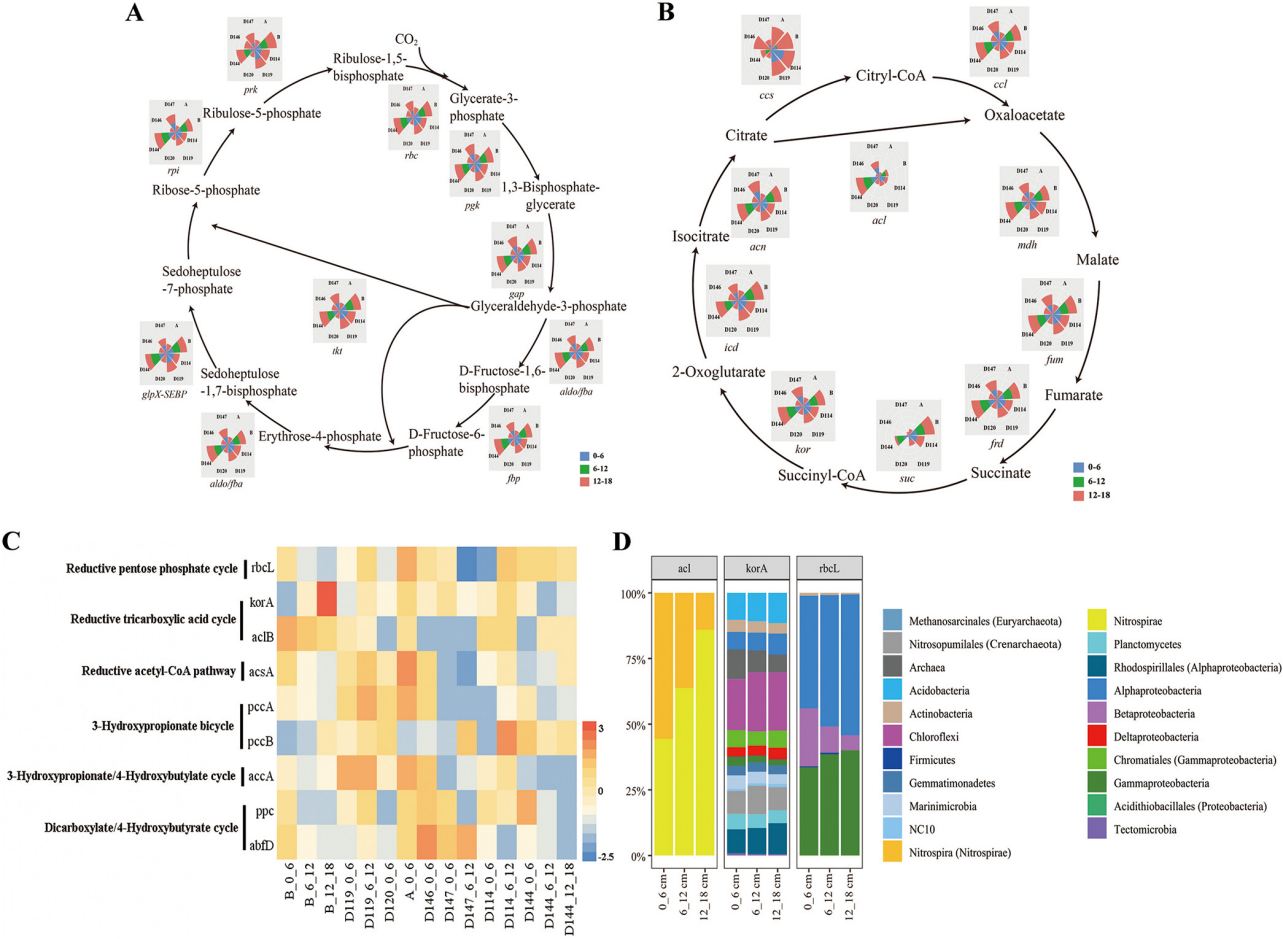
Schematics with pie charts showing the relative abundance of genes involved in the Calvin cycle (A) and rTCA (B) for the metabolism of carbon-fixation in the core microbiome. The abundance was calculated by dividing the sum of the coverage of a particular gene in an individual sample, by the sum of the coverage of that gene in all the samples. (C) The absolute abundance of each gene was shown by z-score heatmap boxes indicated in the Calvin cycle and rTCA cycle. (D) The microbial taxa of typical genes and their relative abundance in the different depths of each region.

The genes involved in six carbon fixation pathways were detected, and all the genes involved in the Calvin cycle (Fig. 7A) and rTCA were present (Fig. 7B). The key *rbc*L gene encoding ribulose-bisphosphate carboxylase in the Calvin cycle was detected (Fig. 7C) and affiliated with microbial taxa of Alpha/Beta/Gammaproteobacteria (Fig. 7D). The *acl*B gene, encoding enzymes catalyzing citrate to oxaloacetate in the rTCA pathway, were generally more abundant in the surface sediment (Fig. 7C) and associated with Nitrospirae. In addition, *kor*A is affiliated with highly diversified microbial taxa, including Acidobacteria, Chloroflexi, Nitrosopumilales, Planctomycetes, Marinimicrobia, Methanosarcinales, NC10 bacteria, and Rhodospirillales (Fig. 7D).

### Taxonomic annotation and metabolic reconstruction of metagenome-assembled genomes (MAGs).

**FIG 8:**
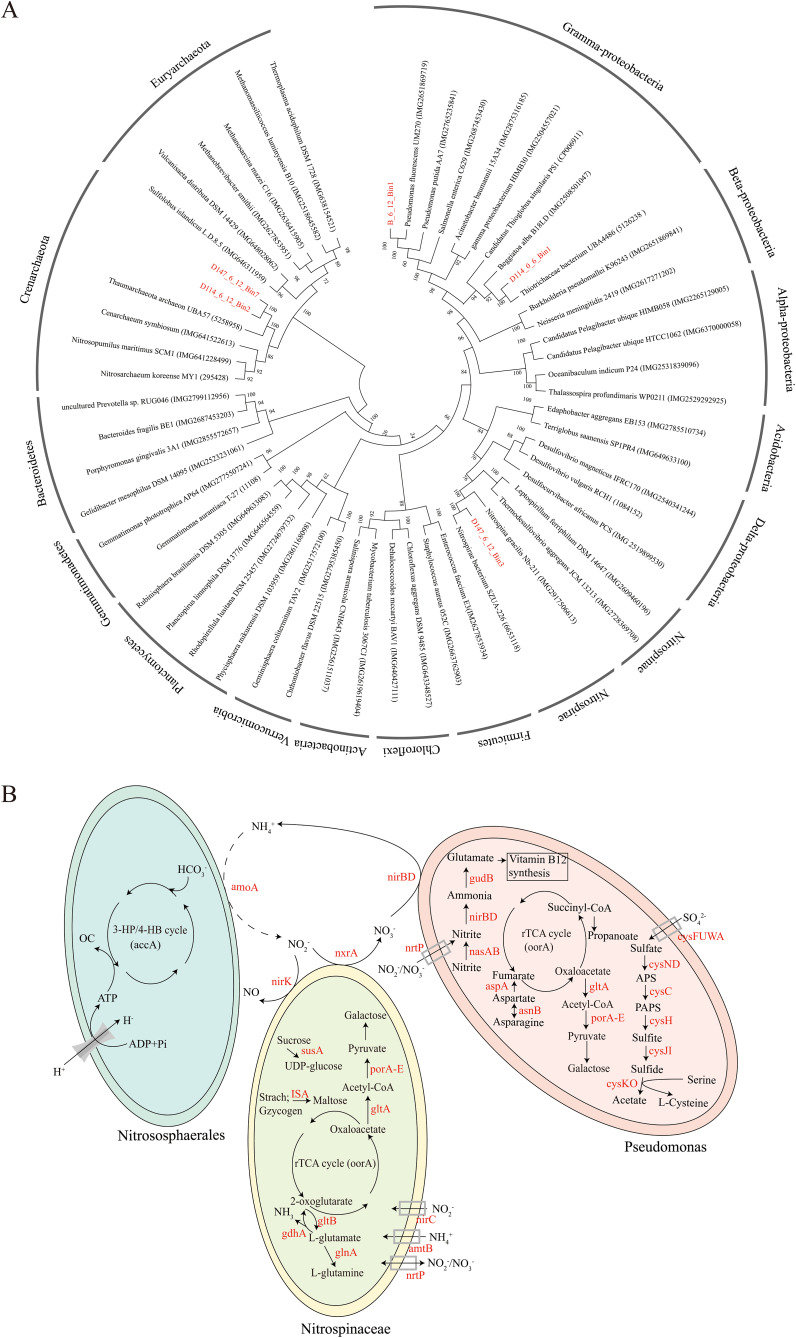
Phylogenetic analysis based on conserved proteins (A) and overview of metabolic potentials (B) of MAGs.

In total, 25 MAGs were obtained from the metagenomic data in this study (Table S1), and five high-quality MAGs (estimated completeness >80%, contamination rate <2.5%) were selected for subsequent analysis. Taxonomic classification based on the phylogenomic tree of concatenated conserved proteins indicated that the MAGs were affiliated with three prokaryotic phyla, including two associated with Crenarchaeota, two related to Pseudomonas and Thiotrichaceae of the Gammaproteobacteria and one affiliated with Nitrospinota (Fig. 8A). Their metabolic potentials in the biogeochemical and nutrient cycling were predicted according to the gene annotation results. The presence of central carbohydrate metabolism, such as tricarboxylic acid cycle, pentose phosphate, and glycolysis pathways was verified in bacterial bin Pseudomonas, which also had the capacity of performing nitrite and sulfur reduction. Key genes (e.g., *acc*A) involved in the 3-HP/4-HB cycle were found in archaeal Bin Nitrososphaerales, which did not contain all the genes necessary for the ammonia oxidation process, probably due to genome incompleteness. Key genes associated with nitrogen metabolic pathways, mainly, including nitrification (e.g., *nxr*A) and nitrite reduction (e.g., *nir*BD), were identified in Bin Nitrospinaceae (Fig. 8b).

## DISCUSSION

### Core microbiome.

The core microbiome represents the collective entities of microorganisms common to most samples and assemblages, and it generally plays an important role in the composition and functions of the microbial community (19). Biogeographic patterns and in particular the relationship between endemism and cosmopolitanism, remain largely unknown for prokaryotic communities in the deep sea. A high level of endemism and a low level of cosmopolitanism of bacterial communities has been revealed in the bathyal and abyssal seafloor of the Pacific Ocean (20), suggesting a limited dispersal of marine benthic bacterial communities. In our study, provincialism due to the limited dispersal caused by the weak deep-water currents (21, 22) was not observed in the marine sediments. Instead, very few bacterial genotypes showed specific distribution, and the core microbiome was composed of several groups of abundant genotypes, indicating a high level of cosmopolitanism of microbial communities along the abyssal-hadal transition zone. This discrepancy might be because our samples were collected from the same trench with less geographical distance, and different statistical analyses for the share and endemic operational taxonomic unit (OTU) were applied as well.

Against the SILVA 128 database, heterotrophic *Woeseia* and *Gemmatimonas* (or Pseudomonas, depending on the depth), and the chemoautotrophic *Nitrosopumilus* accounted for more than 98% of the identified taxa in our core microbiomes. Pseudomonas was the first bacterial assemblage isolated from the sediments of a hadal trench (23, 24). *Woeseia* is suggested to consume the nitrate provided by nitrifiers such as Crenarchaeota (18, 25), and it plays a vital role in coupling carbon and nitrogen metabolism. The co-occurrence of both the heterotrophic bacteria Chloroflexi, Bacteroidetes, Marinimicrobia, Gemmatimonadetes, and Woesearchaeota, Planctomycetes and chemoautotrophic archaea marine group I (MGI) Crenarchaeota have previously been found to dominate the bottom sediments of the Challenger Deep (26).

Those recovered MAGs align well with the dominant microbial groups inferred from metagenomic data, and most reads were of microbial origin based on the calculation of the ratio of prokaryotic to eukaryotic reads (Table 2). Metagenomics could capture microbes with low abundances and unravel the full microbial communities of the studied environmental samples, while MAGs could be reconstructed from the large data set of metagenomics to identify the vertical metabolic functions of the dominant uncultured microorganisms in detail. These two methods have been applied together in different microbial ecological studies conducted in the Mariana Trench (15, 16, 27).

Further analysis of the function of the core microbiome revealed that pathways involved in respiration (e.g., the TCA cycle, NADH: quinone oxidoreductase, heme biosynthesis, pentose phosphate pathway, and IMP biosynthesis) and carbon fixation (e.g., rTCA, 3-HP and DC/HB) were the predominant processes might occur in the abyssal-hadal zone sediment. Genes associated with Erythromycin biosynthesis and *Van*B type vancomycin resistance were found in the surface and middle layers, respectively, which might suggest a potentially stable source of antibiotic resistance gene existed in the sediment core. Distinct microbial community structure and metabolic potentials were found between the waters and sediments in the hadal biosphere at Yap Trench (28) and Marian Trench (26), and the possibility of contamination from water samples could be ruled out. In recent years, biochemical and bioactive diversities of the secondary metabolites, including antibiotics, from the Mariana Trench-sourced microorganisms were discovered in increasing numbers (29). Stress response and metal resistance genes have also been detected in the seawater of the Yap Trench because of the microbial metabolic adaptation to the hadal biosphere (28). The presence of such antibiotic synthesis/resistance genes underscores the fact that deep-sea trenches are a largely unknown resource for novel antibiotics.

### Community composition and environmental effects.

Bacteria were more abundant than archaea in this transition zone, this is consistent with the pattern detected in the seawater and surface sediment in the Yap Trench (28) and suggested that archaea constitute just a small portion of the total microbial community of the detritus-fueled oxic seafloor (30). Anaerobic nitrogen fixers, Rhodospirillales, Rhizobiales, and Chromatiales, were found as major groups of Proteobacteria, consisting of the findings from the Yap Trench (28), as well as previous studies conducted in the Mariana (6, 12, 13) and Puerto Rico (31) Trenches. A niche partitioning for the major archaeal groups was observed in this study, with *Nitrosopumilus* prevalent especially in the surface sediments, and a relatively high abundance of *Nitrosoarchaeum*/*Nitrosotenuis* showed in the deep layers. The prevalence of *Nitrosopumilus* has been detected in the surface sediment in the Yap Trench (28), this was attributed to its capability to outcompete the bacterial counterpart in the oxygen-limited deep-sea sediments (32). In addition, it should be noted that the SILVA database has been updated to v.138 recently, and this might be helpful with better taxonomy classification of the microbial groups, therefore the latest version of the database should be applied in the future study.

Nitrospinales and Nitrospirales present with relatively low abundance in our study, but they were major nitrite-oxidizers in the hadopelagic sediments of the Ogasawara Trench (33). *Nitrospina* can survive in nitrite-depleted oceanic waters (34, 35) and *Nitrospira* is known to prefer lower nitrite concentrations (36). Similar to the finding in the Ogasawara Trench (33), the co-occurrence of the low-substrate/low-oxygen preferring ammonia-oxidizing *Nitrosopumilus* and the nitrite-oxidizing Nitrospinales and Nitrospirales in our study suggests that there might be a complete nitrification pathway available for converting ammonia to nitrate via cooperation between these autotrophic archaea and bacteria in the sediments of this transition zone. Nitrospinae detected from the abyssal-hadal transition zone in our study were not found in the sediment core near the bottom of the Mariana Trench (10,257 m) (26) and the bathyal/abyssal seafloor (20) and might represent specific genotypes in this abyssal-hadal transition zone.

In our study, the most important factors that influenced the spatial variation of the microbial community were the sediment depth and δ^15^N, whereas the geographical distance among the different stations explained very little. The effect of sediment depth was obvious because, along the fine scale of different sediment depths, a clear separation in terms of the microbial composition and average *α*-diversity was revealed. This was consistent with the fact that the microbiome of the surface sediment as being distinct from the subsurface counterpart across the oligotrophic bathyal and abyssal seafloor (20). On the other hand, biogeochemical proxies are used to help disentangle the source of organic matter. For example, marine algae typically have atomic C/N ratios of 4 to 10, and a δ^13^C value of ~−20% (37), compared with δ^13^C values ranging from ~44% to 77% for organic matter from chemosynthesis (38). In our study, the δ^15^N and δ^13^C values suggested that the source of organic matter originated largely from marine algae sinking from the euphotic layer, and together with sediment depth drive the spatial pattern of the microbial diversity in this abyssal-hadal transition zone.

### Ecological roles.

**FIG 9:**
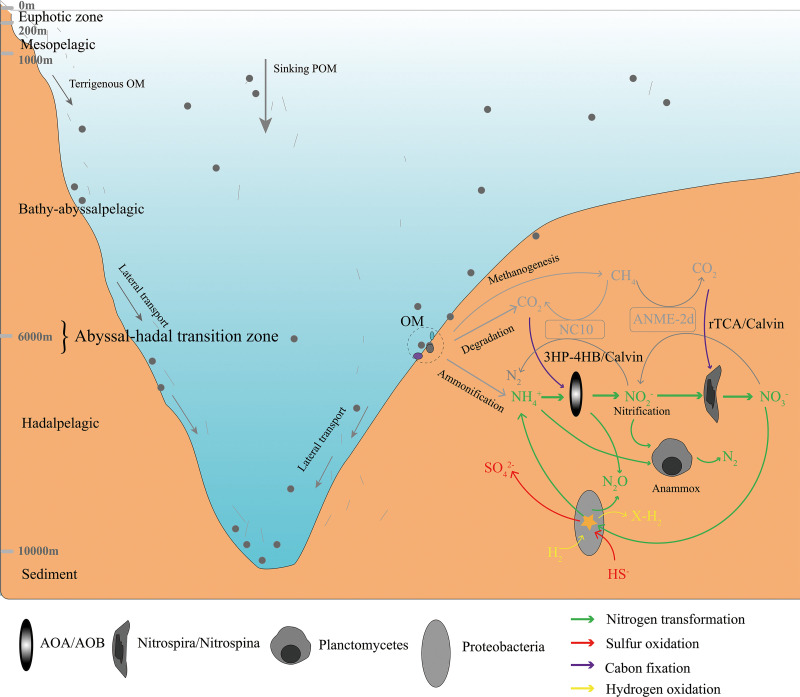
Schematic demonstrating the interactions among the key pathways involved in energy-yielding processes derived from metagenomic information. Heterotrophic remineralization and energetic coupling of nitrogen, sulfur, and hydrogen-based chemotrophic energy metabolism with carbon fixation. Key microbial groups were illustrated, and the associated metabolic pathways are color-coded.

It is known that heterotrophic and chemoautotrophic microbes are predominant in the hadal and abyssal pelagic zones, respectively, possibly driven by the availability and recycling of endogenous organic matter (6, 39). The pelagic vertical stratification of microbial trophic style might not apply to the microbiomes in sediments. We found a predominance of heterotrophic over chemolithoautotrophic pathways, especially for the core metabolism in the abyssal-hadal transition zone (Fig. 9). This suggests that sedimentary organic matter originated largely from sinking marine algae rather than the surrounding inorganic nutrients that might shape the structure and metabolism of the microbial community in this zone. In addition, the diverse metabolic potentials we found with regard to the carbon, nitrogen, and sulfur cycles reflected the lifestyle versatility of the microbial communities that inhabit the abyssal-hadal transition zone, as well as the coupling of the different biogeochemical cycles that occurs in this region (40).

Heterotrophic microbes control the decomposition of organic matter and contribute to the regeneration of nutrients in the ocean, subsequently playing a vital role in favoring shaping the benthic microbial community (6). In our study, typical heterotrophic Bacteroidetes, and Gammaproteobacteria such as Alteromonadales, Oceanospirillales, and Pseudomonadales were abundant, which might drive many fundamental processes, including the oxidation of organic matter, production of methane, and removal of sulfate (12).

Nitrification is believed to be one of the main processes responsible for dark carbon fixation in the ocean interior (41). The spatial distribution of different groups of nitrifiers provides a signature of ammonia flux from organic matter decomposition, and so this can be used as an index of carbon and nitrogen cycling in the oceanic ecosystem (6). In our study, different groups of nitrifiers were identified, and might through respective 3-HP/4-HB and Calvin cycles for ammonia-oxidizing archaea and bacteria (42) and through the Calvin cycle and rTCA cycle for nitrite-oxidizing bacteria, *Nitrospira* and *Nitrobacter*, to fix carbon (43, 44), and potentially contribute to the sustenance of the prokaryotic heterotrophic carbon requirements. This suggested that nitrification was an important way of energy production for sediment microbes in the transition zone. In addition, the potential nitrogen loss processes, anammox, and denitrification were revealed in this study as well. The co-occurrence of aerobic and anaerobic ammonia oxidizers and nitrate reducers (indicating cooperation between aerobic and anaerobic nitrogen metabolism) has previously been revealed in the hadal sediments of the Ogasawara Trench (33), suggesting relatively broad oxic-anoxic gradients for nitrogen metabolism. Both degradation and nitrification are important oxygen-consuming processes. Their co-occurrence might be due to a sequential metabolic reaction, started with a release of ammonia through degradation by heterotrophic microbes and followed by sequential oxidation of ammonia to nitrate by ammonia-oxidizing microbes. Therefore, microbial assemblages involved in the degradation-nitrification processes might have reciprocal interaction via nutrient exchanges.

Organic matter in the hadal trenches can also come from *in situ* dark carbon fixation (chemosynthesis) to meet the microbial carbon demand. Two complete carbon-fixation pathways (the Calvin cycle and rTCA cycle) were detected in our study, representing a potentially autotrophic carbon fixation process to provide additional carbon sources to ecosystems. These are distinct from the intact Calvin cycle and 3-hydroxypropionate/4-hydroxybutyrate (HP/HB) cycle revealed from the sediment of the Yap Trench (28). The Calvin cycle is the most significant carbon fixation pathway on earth, and its presence in the Mariana Trench indicates the intense microbial activity that occurs in this deep-sea biosphere. The rTCA cycle is the predominant carbon fixation pathway in deep-sea vents to facilitate the assimilation of simple organic substrates and has a competitive advantage over obligate lithoautotrophs or heterotrophs (45), therefore, requires significantly less energy for synthesizing organic carbon under anaerobic and reducing environments (46). This metabolic feature might help with the adaptation to deep-sea environments in a similar manner to that found for enriched heavy metal resistance genes in hadal zones (28, 31), mixotrophic features possessed by new ecotypes of Crenarchaeota and SAR11 (47), and the intact HP/HB cycle revealed in the Yap Trench (28).

The deep ocean sediments host diverse microbial communities with important functions in the carbon and nutrient cycles. Our metagenomic data revealed a fine-scale population shift in the different sediment layers and a high level of cosmopolitanism in the core microbiome along with the abyssal-hadal transition zone sediments of the Mariana Trench. Heterotrophic rather than chemolithoautotrophic pathways are predominant over the core metabolism in this transition zone. Information about the nitrogen/carbon-metabolic processes and associated microbes were reflected based on the detection of functional and genetic markers from the metagenomic data in the current study, however, their relative contribution to nitrogen/carbon flux in the abyssal-hadal transition zone still needs further analysis.

## MATERIALS AND METHODS

### Sample collection, genomic DNA extraction, and sequencing.

Sediment push core samples were collected from eight stations along the northern and southern slopes of the Challenger Deep in the Mariana Trench during cruises by DY37II and DY38III in 2016 and 2017, respectively (Fig. 1). *In situ* hydrographical parameters (i.e., the temperature, depth, and location) were also recorded during sampling, using the Jiao Long Human Occupied Vehicle (HOV). The top three layers (i.e., at 0 to 6 cm, 6 to 12 cm, and 12 to 18 cm) of each sediment core were sliced and then immediately stored at –80°C until further analysis.

The sediments were centrifuged to remove any pore water, and then the genomic DNA was extracted using the PowerSoil DNA isolation kit (MO BIO Laboratories, Inc., Carlsbad, USA) according to the manufacturer’s instructions. The DNA was quantified with Qubit 2.0 (Life Technologies, USA), and the quality was checked via gel electrophoresis. The extracted DNA in a total volume of 50 μL in a 0.5 mL microcentrifuge tube was fragmented using covaris adaptive focused acoustics to 350 bp. After end-polished and A-tailed following the standard Illumina protocols, the fragments were ligated with adaptors for Illumina sequencing with further PCR amplification and purification using the NEBNext Ultra II kit (New England Biolabs). After the construction of the library, an Agilent 2100 bioanalyzer system (Agilent Technologies, Santa Clara, CA, USA) was used to detect the inserted size of the library. Sequencing was performed with an Illumina novaseq6000 PE150 platform (Novogene Co., Ltd., www.novogene.com).

### Chemical analysis of the sediments.

A sediment properties analysis with ~10 g of fresh sediment was conducted at the Institute of Mountain Hazards and Environment, Chinese Academy of Sciences (Chengdu, Sichuan, China), according to Wang et al. (48). In brief, nitrate and ammonia were detected after 1 M HCl treatment followed by analysis with a colorimetric Auto-analyzer 3 (SEAL Analytical GmbH, Norderstedt, Germany). The concentrations of total carbon (TC) and total nitrogen (TN) were determined by overdrying the sediments at 105°C and then using a Vario Macro cube element analyzer (Elementar Analysensysteme GmbH, Langenselbold, Germany). Total phosphate (TP) was measured after digestion of the sediment with nitric-perchloric acid (49), using the molybdate colorimetric method with a Shimadzu UV-2450 UV-Visible spectrophotometer (Shimadzu Corp., Kyoto, Japan). Analysis of stable C and N isotopes was conducted in the Nanjing Institute of Geography and Limnology, Chinese Academy of Sciences (Nanjing, Jiangsu, China), using the method described by Liu et al. (50).

### Bioinformatics analysis.

**(i) Metagenome assembly**. The sequencing data of each sample were verified according to their barcode. After being trimmed to remove the adapter sequence, the high-quality sequences (i.e., those of length > 140 bp, without ambiguous base “N,” and with an average base quality > 38) were selected using the FASTX-Toolkit for further analysis (51), and then they were checked using FastQC (Babraham Bioinformatics, Babraham Institute, Cambridge, UK; www.bioinformatics.babraham.ac.uk). All the taxonomic and functional annotations were based exclusively on the quality reads. The number and size of contigs, as well as the GC content and annotated genes, were listed in Table 2. High-quality short reads from each sample were *de novo* assembled using MEGAHIT version 1.2.9 (52) with kmer 55, -d 1, -M 3, -R, -u, -F.

The sequences at the genus level and genes encoding enzymes that occurred in > 90% of the samples were selected to make a “Core table”. The subsequent analysis of both the taxa and function was based on this “Core table” and was constructed with R version 3.5.3.

### (ii) Prokaryotic taxonomic assignment and analysis.

To further confirm the community composition of the core microbiome, the SSU rRNA gene sequence was extracted from the Illumina data using Metaxa2 version 2.2.2 (53). The obtained SSU rRNA reads were trimmed to 450 bp targeting V3 to V4 domains of the 16S rRNA gene and compared against the SILVA 128 database (www.arb-silva.de; (54)) in QIIME for further taxonomic assessment with uclust method following the pipeline analysis described previously (55). In addition, a filtered table of OTUs, showing the alpha and beta diversity of prokaryotes, was generated with QIIME 1.9.1.

### (iii) Functional annotation.

The open reading frames (ORFs) of assembled contigs were predicted by MetaGeneMark version 3.38 (56) with default parameters and then clustered using CD-HIT version 4.6.8 (57) with -c 0.95, -G 0, -aS 0.9, -g 1, -d 0 to remove sequence redundancy and improve the performance of sequence analyses. The clean reads were used to map back to the predicted genes with Bowtie 2.2.9 (58) with –end-to-end, –sensitive, -I 200, -X 400 to get an accurate value for the abundance of each gene. Functional annotations of the assembled scaftigs (unigenes) were performed to hit the KEGG database release 90.1 (https://www.genome.jp/kegg/) (59), the nonredundant protein (NR) database available at NCBI. The blast result of the NR database was also obtained via the Diamond software (60) to identify the taxonomy of each scaftig.

### (iv) Genome binning and annotation.

Assembly and binning were performed individually for each metagenome generated from each sample. Genome binning was performed using MaxBin v2.0 (61) with >1000 bp contigs. The completeness and contamination rate of the MAGs were assessed by CheckM (v1.0.5) (62). Open reading frames (ORFs) and proteins in the MAGs were predicted by Prodigal (v2.6.2) (63). The predicted ORFs were annotated by BLASTP searching against NCBI-nr. The metabolic profiles of MAGs were reconstructed using KEGG (59) and CAZy databases (64). Taxonomic classification of MAGs was obtained using the protein phylogeny in the Genome Taxonomy GTDB 95 Database with the gtdbtk classify_wf command using GTDB-Tk v1.3.0 (65).

About 122 commonly conserved proteins for archaea and 120 for bacteria were identified using hmmsearch (3.0) (66). The reference genomes were downloaded from the NCBI GenBank database, and the alignment of the sequences was performed using MEGA v6.0.6 (67). A phylogenetic tree was constructed by a maximum likelihood (ML) algorithm using MEGA, with the nearest-neighbor-interchange (NNI) method for 1000 bootstrap iterations.

### Statistical analysis.

The nonlinear multidimensional scaling (nMDS) based on the Bray-Curtis similarity index was calculated with PRIMER 5 (Plymouth Marine Laboratory, West Hoe, Plymouth, UK) (68). Redundancy analysis (RDA) was performed to identify a possible differentiation of the communities under the constraint of environmental factors and assess correlations between environmental variables and community variability. To avoid collinearity among environmental variables, high variance inflation factors (i.e., VIF > 10) were eliminated. The RDA and heatmap of genes coding enzymes involved in the nitrogen, sulfur, or carbon fixation metabolic pathways were performed using the vegan (69) and “ggplot2” packages in R version 3.5.3, respectively. The absolute abundance of each gene was normalized to z-scores. A permutational multivariate analysis of variance (i.e., the ANOSIM test) was conducted to determine whether there was a significant difference in the microbial communities among the various sampling sites based on Bray-Curtis similarity with paleontological statistics (PAST) version 3 (70).

### Data availability.

The raw sequence data for metagenomics have been deposited in the National Center for Biotechnology Information (NCBI) Sequence Read Archive (SRA) under accession number PRJNA629672. The sequence data for MAGs from the sediment of Mariana Trench have been deposited in the National Genomics Data Center, Beijing Institute of Genomics (China National Center for Bioinformation), Chinese Academy of Sciences, under accession number PRJCA009742.
