# Supplementary material for: Composition and Ecological Roles of the Core Microbiome along the Abyssal-Hadal Transition Zone Sediments of the Mariana Trench
Source: Microbiol Spectr. 2022 Jun 7;10(3):e01988-21. doi: 10.1128/spectrum.01988-21 (PMC9241748; doi:10.1128/spectrum.01988-21)
Supplement: SUPPLEMENTAL FILE 1 — Supplemental material. Download spectrum.01988-21-s0001.pdf, PDF file, 0.2 MB [file spectrum.01988-21-s0001.pdf]

Supplementary materials:

Table S1 General characteristics of metagenome-assembled genomes (MAGs) of the sediment samples collected from the Challenger Deep in the Mariana Trench.

Sample	Completeness	Contamination	GC	Coverage	Taxonomy
B_0_6_Bin1	83.37	4.63	60.52	84.1	Methylomirabilota; Methylomirabilales
B_0_6_Bin2	87.64	14.29	60.09	82.2	Acidobacteriota; Acidobacteriae
B_6_12_Bin1	98.47	0.38	61.14	67.3	γ-proteobacteria; Pseudomona
B_12_18_Bin1	92.62	20.13	59.40	89.7	Methylomirabilota; Methylomirabilales
B_12_18_Bin2	76.19	21.70	60.22	57.0	γ-proteobacteria; Acidiferrobacterales
B_12_18_Bin3	75.28	14.54	61.53	71.0	γ-proteobacteria; Pseudomonadales
D114_0_6_Bin1	80.09	0.82	53.05	86.9	γ-proteobacteria; UBA4486
D114_0_6_Bin2	76.14	22.19	46.18	27.1	Thermoproteota; Nitrososphaerales
D114_6_12_Bin1	78.1	23.72	55.30	68.2	Nitrospinota; Nitrospinaceae
D114_6_12_Bin2	82.30	0.00	46.31	28.0	Thermoproteota; Nitrososphaerales
D119_6_12_Bin1	76.11	11.54	66.35	58.9	Myxococcota; SZUA-336
D119_6_12_Bin2	95.53	14.14	54.09	93.5	γ-proteobacteria; Acidiferrobacterales
D120_0_6_Bin1	94.83	16.69	53.87	95.3	γ-proteobacteria; Acidiferrobacterales
D144_12_18_Bin1	77.64	12.02	61.51	77.6	γ-proteobacteria; Pseudomonadales
D144_6_12_Bin1	86.61	13.77	61.45	81.3	Actinobacteriota; Acidimicrobiia
D144_6_12_Bin2	75.29	16.30	63.21	65.4	Gemmatimonadota; Gemmatimonadetes
D147_0_6_Bin1	75.04	23.96	65.67	49.5	Gemmatimonadota; Gemmatimonadales
D147_6_12_Bin1	83.45	23.37	58.26	81.3	Gemmatimonadota; Gemmatimonadales
D147_6_12_Bin2	78.31	23.62	65.62	70.1	Gemmatimonadota; Gemmatimonadales
D147_6_12_Bin3	83.33	0.05	54.66	84.1	Nitrospinota; Nitrospinaceae
D147_6_12_Bin4	92.48	14.60	57.44	82.2	Gemmatimonadota; Gemmatimonadales
D147_6_12_Bin5	92.24	13.79	55.51	93.5	γ-proteobacteria; Woeseiaceae
D147_6_12_Bin6	81.57	21.16	52.12	78.5	Bacteroidota; Rhodothermales

D147_6_12_Bin7	92.25	2.02	46.06	21.5	Thermoproteota; Nitrososphaerales
D147_6_12_Bin8	77.21	22.73	60.59	62.6	Planctomycetota; Pirellulales

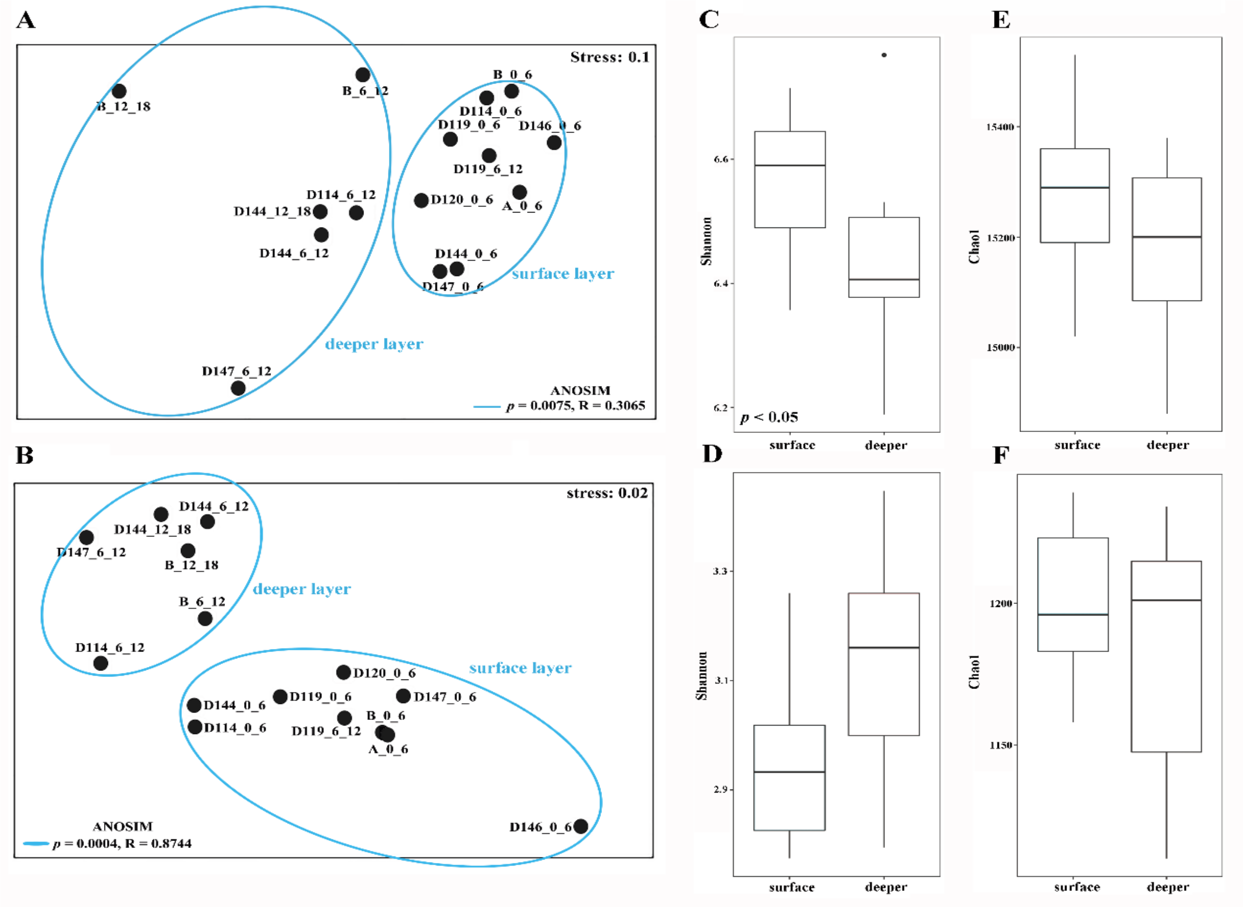

Figure S1 NMDS plot and averaged α-diversity indices at the order level for the bacterial (A, C and E) and archaeal (B, D and F) assemblages.
